# Weaning from mechanical ventilation in myasthenic crisis according to WEAN safe: most patients experience intermediate or prolonged weaning with no differences between early and late-onset compared to very-late onset myasthenia Gravis

**DOI:** 10.1186/s13613-025-01515-2

**Published:** 2025-07-14

**Authors:** Clémence Marois, Arthur Combes, Meriem Bouguerra, Alexandra Grinea, Lucas Di Meglio, Thomas Rambaud, Loïc Le Guennec, Francis Bolgert, Benjamin Rohaut, Sophie Demeret, Nicolas Weiss

**Affiliations:** 1 Sorbonne Université, AP-HP.Sorbonne Université, Hôpital de la Pitié-Salpêtrière, Médecine Intensive Réanimation à orientation neurologique, Paris, France; 2 Groupe de Recherche Clinique en REanimation et Soins intensifs du Patient en Insuffisance Respiratoire aiguE (GRC-RESPIRE) Sorbonne Université, Paris, France; 3 Institut Cochin, Université Paris Cité, CNRS, Inserm, Paris, France; 4 Paris Brain Institute-ICM, PICNIC Lab, Inserm U1127, CNRS UMR 7225, Sorbonne Universite, Paris, 75013 France; 5 Brain Liver Pitié-Salpêtrière (BLIPS) Study Group, Centre de recherche Saint-Antoine, Maladies métaboliques, biliaires et fibro-inflammatoire du foie, Institute of Cardiometabolism and Nutrition (ICAN), Inserm UMR_S 938, Paris, France

**Keywords:** Myasthenia gravis, Myasthenic crisis, Weaning, Mechanical ventilation

## Abstract

**Background:**

Myasthenic crisis often requires prolonged mechanical ventilation and complex weaning, yet data remain scarce. The objective of this study was to describe the weaning characteristics in patients with myasthenic crisis using the WEAN Safe classification. Secondary aims included assessment of long-term outcome and comparison between early- and late-onset (< 65 years) versus very-late-onset MG (≥ 65 years) myasthenia gravis.

**Methods:**

This single-center retrospective study included patients admitted for myasthenic crisis to a tertiary neuro–intensive care unit between January 2008 and December 2023. Clinical characteristics, ventilatory support parameters, timing of weaning events, complications, and outcomes were recorded. Weaning was classified according to WEAN Safe definitions: no separation attempt, short wean (successful weaning within 1 day), intermediate wean (2–6 days), prolonged wean (≥ 7 days), or failed wean (persistent invasive ventilation at discharge or death).

**Results:**

Among 698 ICU hospitalizations (405 patients) for myasthenia gravis, 131 (120 patients) received invasive mechanical ventilation. Fifty hospitalizations (39 patients) were excluded due to non-MC-related intubation, insufficient weaning data or patients with multiple ICU admissions. The final analysis included 81 patients (median age 70 years [54–81]; 43% female; 64% with very-late-onset myasthenia gravis (≥ 65 years). The median duration of mechanical ventilation was 20 days [11–38], and the median time from the first separation attempt to successful weaning was 7 days [3–19]. According to the WEAN Safe classification, 3% had a short wean, 40% intermediate, 55% prolonged, and 3% failed weaning. Four patients (5%) required reintubation within 48 h. Ventilator-associated pneumonia occurred in 15% of patients before the first separation attempt. In multivariate analysis, the presence of thymoma (OR 3.02, 95% CI 1.01–9.07) and absence of MG-specific immunosuppressive treatment at ICU admission (OR 3.70, 95% CI 1.22–11.23) were independently associated with prolonged weaning. Intensive care unit mortality was 7%, and 12-month mortality was 19%. The median myasthenic muscle score at 1 year was 94/100 [80–100]. No significant differences in weaning parameters nor outcome were found between early- and late-onset versus very-late-onset MG, despite more comorbidities in the very-late-onset group.

**Conclusions:**

In this retrospective study from a single expert center, most patients with myasthenic crisis underwent intermediate or prolonged weaning, but extubation failure rate was very low. Thymoma and lack of MG-specific immunosuppressive treatment at ICU admission are associated with prolonged weaning, while age alone is not. Despite initial challenges, long-term outcomes are generally favorable, highlighting the reversibility of myasthenic crisis with expert care.

## Introduction

Myasthenia gravis (MG) is a rare autoimmune disease with an annual incidence of 8 to 10 cases per million individuals. It affects the neuromuscular junction, leading to varying degrees of muscle weakness [1, 2]. In generalized MG, approximately 80% of patients have antibodies against the acetylcholine receptor (AChR), around 10% against muscle-specific-kinase (MuSK) or other rarer targets and about 10% are seronegative. The age of onset follows a triphasic pattern: (1) early-onset MG (< 50 years) predominantly affects women (female-to-male ratio 3:1) and is often associated with thymic hyperplasia (2) late-onset MG (between 50 and 64 years) shows a slight male predominance (male-to-female ratio 1.5:1) (3) very-late-onset MG (≥ 65 years) is the most common presentation, typically associated with a higher presence of AChR antibodies and fewer thymomas [3]. Thymoma is present in about 10% of generalized MG cases but may be found in up to 30% of MG patients requiring ICU admission [4]. The most severe complication of MG is myasthenic crisis (MC) which is characterized by severe weakness of respiratory and upper airway muscles, necessitating ICU admission for mechanical ventilation (MV) [5]. This life-threatening condition occurs in 15–20% of patients with generalized MG [5] and may represent the initial manifestation or result from disease exacerbation triggered by infections, medication adjustments, non-adherence, or surgical procedures.

Despite being extensively discussed, robust data on the weaning process in MC remain scarce [5–7]. Reported extubation failure (EF) rates in MC reach 43% compared to 13% in the general ICU population [8]. Similarly, the rate of weaning failure (defined as EF, death during intubation or tracheotomy without extubation attempt) can reach 64.2% in MC patients [9], compared to 35% among ICU patients ventilated for other reasons [8, 10]. Notably, late-onset MG appears to be more common among patients requiring prolonged ventilation [9].

The WEAN Safe study is a recent international, multicenter, observational, prospective study involving over 5,000 ICU patients from more than 50 countries [10]. It described weaning delays, timings of weaning events, associated risk factors, and outcomes; and proposed a classification system for weaning profiles. However non-traumatic neurological conditions represented only 14.4% of admissions, and data specific to neuromuscular disorders, particularly MG, were not included.

The aims of our study were (1) describe the management, timing, and outcomes of weaning in MC patients using the WEAN Safe classification, (2) report long-term neurological outcome and (3) compare early- and late-onset (< 65 years) with very-late-onset MG (**≥** 65 years).

## Materials and methods

### Study design

We conducted a retrospective study including all patients admitted for MC to the Neuro-ICU at La Pitié-Salpêtrière University Hospital, a tertiary care referral center in Paris, between January 2008 and December 2023. The hospital serves as a national reference center for neuromuscular diseases, including MG.

### Ethics

The study was approved by the ethics committee of the “*Société de Réanimation de Langue Francaise*” (CE SRLF 23–081). All procedures followed the ethical standards of the institutional and national research committees and the principles of the Declaration of Helsinki (1975).

### Patient selection

All patient with a diagnosis of MG who received invasive mechanical ventilation during their ICU stay were identified using the institutional information Systems Medicalization Program and relevant procedure codes (G700 or G730 and GLLD004, GLLD008 or GLLD015 from the French Common Classification of Medical Procedures). Patients were excluded if they were intubated for reasons unrelated to MC or if data on the weaning process were incomplete or if they had an ICU admission within a 3-months interval for a cause other than a MC. In the case of recurrent MC, only the first MC was included in the analysis. MC was defined by severe weakness of respiratory and upper airway muscles, necessitating MV or delaying extubation after surgery [5].

### Definitions

A spontaneous breathing trial (SBT) was defined as a period of reduced or absent ventilator support aimed to predict weaning success.

The weaning process was characterized based on the WEAN Safe study definitions [10]. Separation attempt (SA): either an SBT or a direct extubation without prior SBT in intubated patient, or on SBT in tracheostomized patient.

Weaning success in intubated patient was defined as extubation without death or reintubation within 7 days, or ICU discharge without invasive ventilation. In tracheostomized patients, weaning success was defined as spontaneous breathing during at least 7 consecutive days. Weaning failure was defined as persistent invasive ventilation at day 90, death before weaning success, or transfer to another unit while still receiving invasive mechanical ventilation.

Extubation failure (EF) was defined as reintubation or death within 7 days of the extubation. Patients who experienced extubation failure but were subsequently successfully weaned were not classified as weaning failures.

In contrast to the definitions used in the WIND and WEAN SAFE studies, we chose to exclude unplanned extubations from SA and reintubation following such event was not categorized as EF. This decision was based on the specific context of myasthenic crisis, where unplanned extubations are generally not reflective of a structured weaning process. Notably, previous studies in this population [9, 11] did not consider unplanned extubation as separation attempts either.

WEAN Safe classification groups: (1) No SA: no SA before death or transfer (2) Short wean: weaning success within 1 day of the first SA (3) Intermediate wean: success within 2–6 days (4) Prolonged wean: success at least 7 days after the first SA and (5) Failed wean: persistent invasive ventilation at day 90, transfer out of the ICU with ongoing ventilation, or death.

MG onset classification followed previously published criteria [3]: Early-onset: <50 years; late-onset: 50–64 years and very-late-onset ≥ 65 years.

Ventilator-associated pneumonia (VAP) was defined according international consensus definitions, as pneumonia that arises more than 48 h after endotracheal intubation, with new or progressive infiltrates on chest imaging, plus two or more clinical criteria (e.g., fever, leukocytosis, purulent tracheal secretions, hypoxia), supported by microbiological confirmation when available [12].

### Local weaning protocol

As part of an expert center in the field of neuromuscular diseases, we have utilized specific weaning procedures for myasthenic patients during the study period. The SBT begun when (1) classical weaning criteria were fulfilled [8] (hemodynamic stability, absence of fever or sepsis, adequate oxygenation, normal PaCO₂, respiratory rate ≤ 30 breaths/min, tidal volume ≥ 5 mL/kg of ideal body weight, and no ongoing sedation with a normal level of consciousness), (2) the cause of MC has resolved, (3) the patient had at least started immunomodulatory treatment, (4) myasthenic symptoms improved, notably the ability to lift the head and elbows off the bed and to cough effectively, with minimal air way secretions [5].

Two types of SBTs were used: either a zero end-expiratory pressure (ZEEP) trial, in which the patient remained connected to the ventilator with minimal respiratory support (positive expiratory pression = 0 cm H_2_O and pressure support = 7 cm H_2_O); or a T-tube trial, where the patient was disconnected from the ventilator and received supplemental oxygen as needed, without inspiratory support [13]. T-tube trials were performed using both inflated or deflated cuffs to assess swallowing and airway protection [14–18].

In intubated patients, the duration of SBT was gradually increased over several days, based on patient tolerance. It typically began with trials lasting 30 to 60 min and were extended up to 2 to 12 h. The choice of SBT type, trial duration, and the final decision of extubation were made at the discretion of the attending intensivist.

Tracheostomy was performed early, if the expected duration of ventilation exceeded 10–15 days (e.g., severe crisis, uncontrolled triggering) particularly in patients with severe bulbar dysfunction or cough weakness, in order to facilitate weaning and improve patient comfort [19]. Tracheostomy could be performed before any SBT and often preceded extubation attempts. In our center, tracheotomy is usually performed percutaneously, except in cases of contraindications or technical difficulties, in which case it is performed surgically. Initial SBTs on tracheotomy followed the same protocol as for endotracheal tube, followed by T-tube trials with a deflated cuff of increasing duration, ultimately reaching 24 h of continuous spontaneous breathing. Use of a speaking valve or capped cannula was encouraged to promote phonation during the day. If this phase was well tolerated, it was followed by 24 h with a fully capped cannula (i.e., no airflow through the tracheostomy), which constituted the final validation of spontaneous breathing capacity and airway protection prior to proceeding to decannulation.

### Data collection

Data were retrospectively extracted from medical records including: age, gender, comorbidities, MG characteristics (age of onset, disease duration, antibody status, thymus hyperplasia, thymoma, thymectomy), MG treatments (acetylcholine esterase inhibitors, corticosteroids, immunosuppressive drugs), causes of MC (inaugural crisis, infection, therapeutic non-compliance or modification, post-operative or post-procedure, unknown cause), myasthenic muscle score (MMS) at ICU admission, ICU discharge, 6 and 12 months [20], crisis-specific treatments (corticosteroids, IV immunoglobulins (IVIg), therapeutic plasma exchange), reason for intubation (acute hypoxemic respiratory failure defined as clinical signs of respiratory distress with SpO₂ < 90% or requiring oxygen to maintain SpO₂ >90% without elevated PaCO2; acute hypercapnic respiratory failure defined as clinical signs associated with PaCO₂ >45 mmHg with or without concurrent hypoxemia, other indication defined as clinical signs not meeting the previous criteria), Simplified Acute Physiology Score II (SAPS-2) at admission [14], mechanical ventilation metrics (duration of MV, length of stay (LOS) in ICU and in hospital), ICU complications (VAP occurring before the first spontaneous breathing attempt, other infections, hemorrhagic and thromboembolic events, cardiogenic pulmonary edema, acute kidney injury), outcomes (mortality in ICU and at 12 months). Weaning-specific data collected included: date and number of SBTs, characteristics of the final SBT before extubation in intubated patients (duration, type of trial (ZEEP, T-tube, inflated or deflated cuff, if applicable), timing and cause of reintubation, date of tracheostomy.

### Statistical analysis

Continuous variables were expressed as median and interquartile range. Categorical variables were expressed as frequencies and percentages. Chi-square or Fisher exact test were used to compare categorical variables and ANOVA for continuous variables. A multivariate analysis was conducted using forward stepwise logistic regression to identify independent predictors of prolonged weaning (duration between the first SA to successful weaning exceeding 15 days). The following variables were included based on prior literature indicating their potential association with prolonged ventilation [9, 11, 21, 22]: age, presence of cardiovascular comorbidity, presence of thymoma, absence of MG-specific immunosuppressive treatment at ICU admission, presence of acute hypercapnic respiratory failure, infectious cause of myasthenic decompensation and occurence of ventilator-associated pneumonia before the first SA. All statistical tests were two-tailed. P values that were less than 0.05 were considered to indicate statistical significance. Analysis involved the use of JMP Pro v16.0 (SAS Inst., Cary, NC).

## Results

**Fig. 1:**
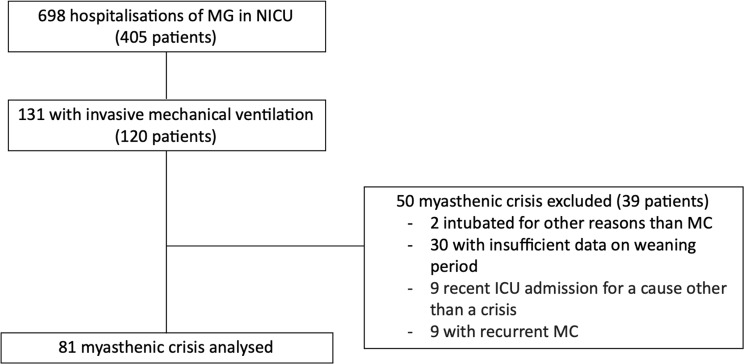
Flowchart Abbreviations: MG: myasthenia gravis, NICU: Neuro- intensive Care Unit, MC: myasthenic crisis,

Between January 1, 2008, and December 31, 2023, a total of 698 ICU hospitalizations involving 405 patients with MG were recorded. Among them, 131 ICU admissions (involving 120 patients) received invasive mechanical ventilation. Fifty hospitalizations (39 patients) were excluded (2 intubated for other reasons than MC, 30 with insufficient data on weaning process, 9 involved prior recent ICU admission within a 3-months interval for a cause other than a MC, 9 with recurrent MC). Ultimately, 81 patients with MC were included in the analysis (Fig. 1).

### Patient characteristics

**Fig. 2:**
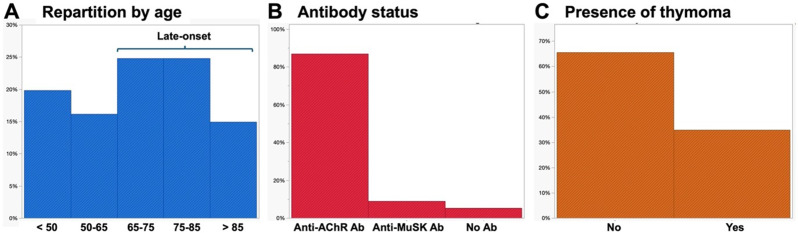
Patients characteristics Main patient’s characteristics are presented: **A**, repartition by age, **B**, antibody status, **C**, the presence or the absence of thymoma

Baseline characteristics of the ICU admission for each of the 81 patients are presented in Table 1. Thirty-five patients (43%) were female (Fig. 2A) with a median age of 70 years [54–81]. Fifty-two (64%) had very late-onset MG; 35% had a thymoma, and 72% had at least one comorbidity; seventy patients (86%) had antibodies against AChR, 7 (9%) against MuSK and 4 (5%) were seronegative (Fig. 2B). Only 40 patients (49%) were receiving immunomodulatory treatment at ICU admission. Myasthenia muscle score at ICU admission was 45 [27–52]. During the ICU stay, 81% of patients were treated with IVIg, and 27% received therapeutic plasma exchange, either alone or following IVIg failure. The reasons for intubation were acute hypoxemic respiratory failure in 42 (52%) patients, acute hypercapnic respiratory failure in 27 (33%) (including 13 (48%) with associated hypoxemia) and other indications in 12 patients (15%).

**Table 1:** Patients characteristics

	*n* = 81
***General characteristics***	
Age, years	70 [54–81]
Female, n (%)	35 (43%)
SAPS-2	34 [26–46]
Comorbidities, n (%)	
None	23 (28%)
Pulmonary disease	15 (19%)
Cardiac disease	46 (57%)
Renal insufficiency	3 (5%)
Liver disease	3 (4%)
Neoplasm (other than thymoma and skin carcinomas)	5 (7%)
Neurological disease	11 (18%)
Other	8 (10%)
***Myasthenia gravis***	
Disease duration, months	12 [2–89]
Age of onset n (%)	
Early	16 (20%)
Late	13 (16%)
Very-late	52 (64%)
Antibody status, n (%)	
AChR	70 (86%)
MuSK	7 (9%)
Seronegative	4 (5%)
Thymoma, n (%)	28 (35%)
Thymectomy, n (%)	22 (27%)
Anticholinesterase treatment, n (%)	63 (78%)
Immunomodulatory treatment, n (%)	
None	41 (51%)
Corticosteroids	32 (40%)
Azathioprine	21 (26%)
Mycofenolate mofetil	6 (7%)
Rituximab	2 (2%)
Cyclosporine	3 (4%)
***ICU admission***	
Provenance/Origin	
MICU/emergency departement	19 (23%)
Hospital ward	23 (28%)
Home	8 (10%)
Other ICU	28 (35%)
Other	3 (4%)
Causes of myasthenic crisis*	
First manifestation of MG / inaugural crisis	26 (32%)
Infection	32 (40%)
Pulmonary	13 (40%)
Bacteria	4 (31%)
Virus	2 (15%)
No positive culture	7 (54%)
Urinary	1 (3%)
Unknown origin	18 (57%)
Therapeutic non-compliance or modification	14 (17%)
Post-operative or post-procedure	10 (12%)
None	16 (20%)
Myasthenic score upon ICU admission, *n* = 58	45 [27–52]
Myasthenic crisis specific treatment, n (%)	
Prednisone (dose ≥ 0.5 mg/kg)	56 (69%)
IVIg	66 (81%)
Therapeutic plasma exchange	22 (27%)

*one patient can have more than one identified causes

Abbreviations: Ach-R: Acetylcholine Receptor Antibody; MuSK Muscle Specific Kinase; ICU: intensive care Unit; IVIg: Intravenous Immunoglobulin; MICU: mobile inbtensive care unit; SAPS-2, simplified acute physiology score

### Ventilation and weaning

**Fig. 3:**
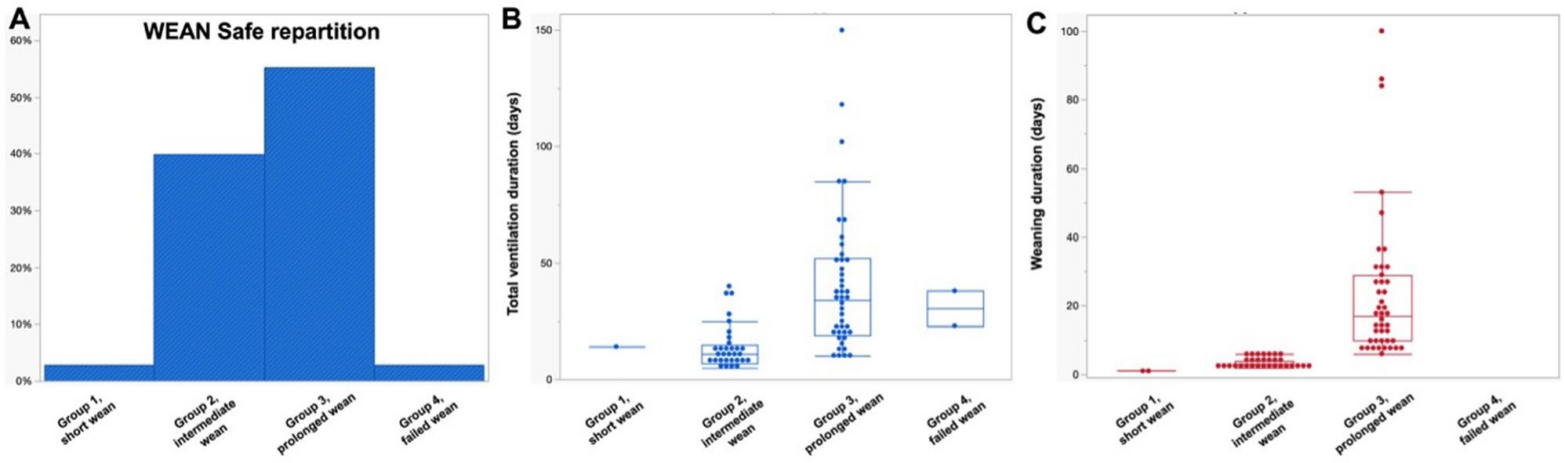
Ventilation and weaning duration according to the WEAN safe classification **A**, WEAN Safe repartition; **B**, total ventilation duration according to WEAN Safe, **C** weaning duration according to WEAN Safe

The median duration of mechanical ventilation for the entire cohort was 20 days [11–38] and the median time from the first SA to successful weaning was 7 days [3–19]. The distribution according to WEAN Safe groups is shown in Table 2; Fig. 3A.

**Table 2:** Ventilation and weaning modalities

	Total population *n* = 81	Intubated patients* n = 47	Tracheotomised patients *n* = 34	*p* value
Reason for intubation, *n* (%)				
Acute hypoxemic respiratory failure, n (%)	42 (52%)	23 (49%)	19 (56%)	0.674
Acute hypercapnic respiratory failure, n (%)	27 (33%)	17 (36%)	10 (29%)	0.438
*With associated hypoxemia*,* n (%)*^$^	*13 (48%)*	*8 (47%)*	*5 (50%)*	-
Other indications, n (%)	12 (15%)	7 (15%)	5 (15%)	-
**Ventilation characteristics**,** days**				
Time from admission to intubation (days)	4 [0–6]	3 [0–6]	4 [0–7]	0.964
Time from intubation to first separation attempt (days)	9 [4–21]	8 [3–12]	12 [6–32]	**0.011**
Time from intubation to tracheotomy (days)	na	na	13 [8–23]	-
Time from tracheotomy to first separation attempt (days)	na	na	8 [4–16]	-
Time from first separation attempt to successful weaning (days)	7 [3–19]	4 [3–7]	20 [10–32]	**< 0.001**
Total ventilation (days)	20 [11–38]	13 [8–19]	38 [23–59]	**< 0.001**
Repeated SBT	72 (89%)	41 (87%)	31 (91%)	0.742
Non-invasive ventilation after extubation, n (%)	11 (14%)	11 (23%)	na	-
Reintubation within 48 h, n (%)	4 (5%)	1 (2%)	3 (9%)	**< 0.001**
Reintubation between 48 h and 7 days, n (%)	0	0	0	-
Final SBT modalities before extubation**	*n* = 45	*n* = 42	*n* = 3	
Final Spontaneous breathing trial, n (%)				**0.005**
T-piece trial	36 (80%)	36 (86%)	0	
T-piece trial with deflated cuff	29 (81%)	29 (69%)	0	
T-piece trial with inflated cuff	6 (17%)	6 (14%)	0	
T-piece trial without cuff data	1 (2%)	1 (2%)	0	
ZEEP trial	9 (20%)	6 (14%)	3 (100%)	
Duration (minutes)	175 [64–475]	180 [85–480]	30 [30–30]	0.182
Duration > 2 h, n (%)	23 (28%)	23 (49%)	0	0.036
WEAN Safe classification, *n* = 78				**< 0.001**
No separation attempt	0	0	0	
Short wean (< 24 h)	2 (3%)	2 (5%)	0	
Intermediate wean (2–6 days)	31 (40%)	28 (64%)	3 (9%)	
Prolonged wean (≥ 7 days)	43 (55%)	14 (32%)	29 (85%)	
Failed wean	2 (3%)	0	2 (6%)	
**Complications during ICU stay**				
Ventilator-associated pneumonia before first spontaneous breathing attempt, n (%)				0.134
0	69 (85%)	43 (91%)	26 (76%)	
1	11 (14%)	4 (9%)	7 (21%)	
2	1 (1%)	0	1 (3%)	
Other infections, n (%)	19 (23%)	10 (21%)	9 (26%)	0.134
Hemorrhagic event, n (%)	3 (4%)	2 (4%)	1 (3%)	0.971
Thromboembolic event, n (%)	7 (9%)	1 (2%)	6 (18%)	0.016
Cardiogenic pulmonary edema, n (%)	7 (9%)	6 (13%)	1 (3%)	0.212
Acute kidney injury, n (%)	7 (9%)	4 (9%)	3 (9%)	0.679
**Outcome**				
Length of stay in ICU, days	32 [22–56]	24 [18–39]	56 [32–83]	**< 0.001**
Length of stay in Hospital, days	41 [27–65]	31 [20–42]	63 [42–94]	**< 0.001**
Death in ICU, n (%)	6 (7%)	3 (6%)	3 (9%)	0.679
Death at 12 months, n (%)	15 (19%)	8 (17%)	7 (21%)	0.683
Myasthenic score at ICU discharge, *n* = 61	80 [66–90]	80 [66–92]	80 [65–87]	0.815
Myasthenic score at 6 months, *n* = 53	90 [82–97]	90 [81–97]	90 [83–98]	0.985
Myasthenic score at 12 months, *n* = 52	94 [80–100]	92 [80–100]	95 [86–99]	0.439

* only were considered patients that were not secondarily tracheotomized. $ corresponds to the number of hypoxemic patients among the hypercapnic patients. ** Final SBT modalities are reported for patients extubated after an SBT and for three tracheotomized patients who were initially extubated but required reintubation and subsequent tracheostomy

**Fig. 4:**
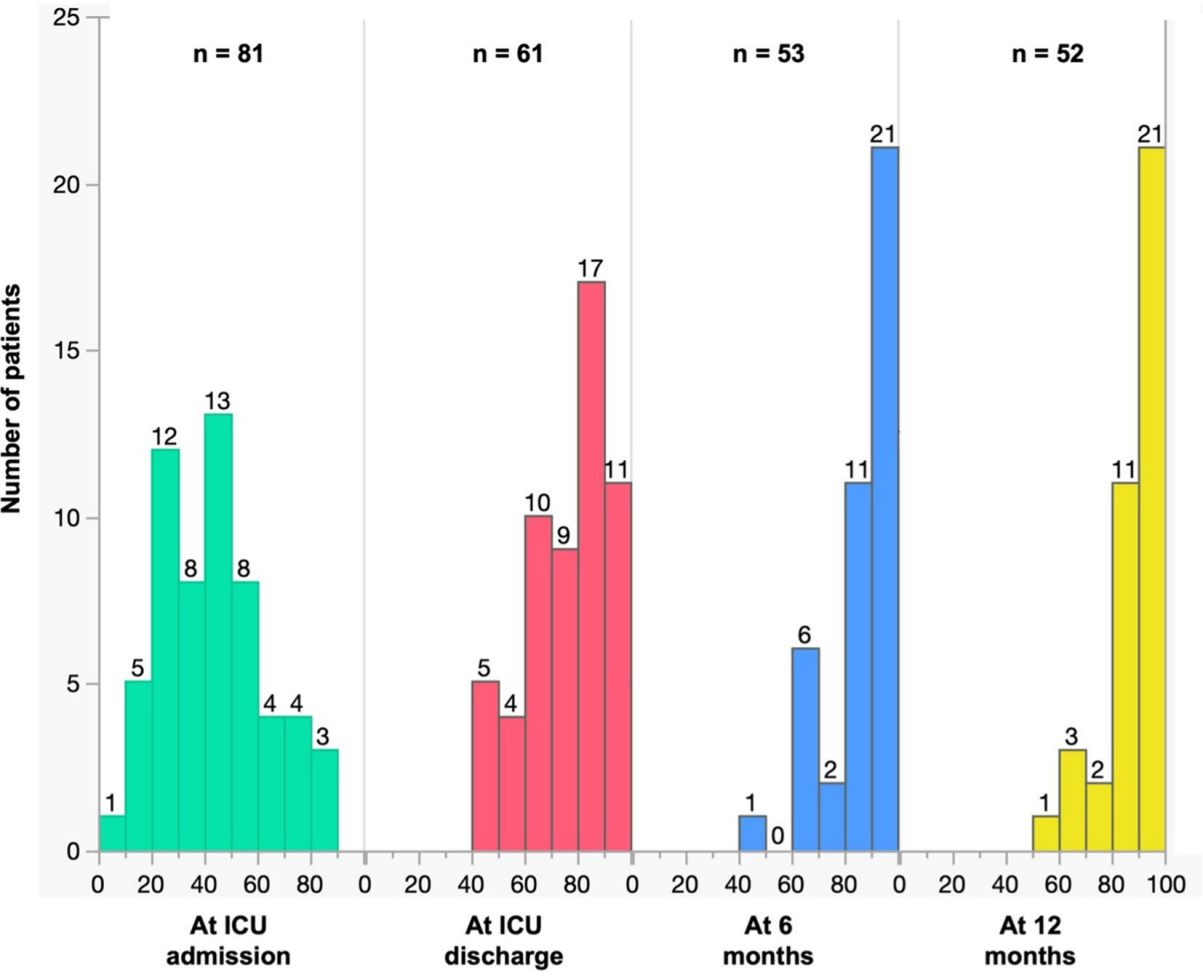
MG score evolution over time Myasthenia gravis mMuscle Score evolution over time. The muscle score is going from 0 to 100, a maximal score of 100 indicates the absence of any symptoms. The numbers above the columns indicated the number of patients that display this score

None of the patients was in the no SA group, 2 patients (2%) were in the short wean group (≤ 1 day), 31 (38%) in the intermediate wean group (2–6 days), 43 (53%) in the prolonged wean group (≥ 7 days), and 2 (2%) in the failed wean group (death before achieving weaning success). Differences between groups in terms of weaning and complications are detailed Table 3; Fig. 3. Forty-seven (58%) intubated patients were extubated without ever requiring a tracheostomy, while 34 (42%) underwent tracheostomy (Table 2). Four patients (5%) required reintubation within 48 h. As expected, total ventilation duration, the duration between first SA and successful weaning, ICU and hospital length of stay varied significantly between groups (Fig. 4). Patients in the prolonged wean group were less likely to have received immunomodulatory treatment for MG at ICU admission compared to the intermediate group (*p* = 0.0044). Final SBTs were mainly performed using T-piece trials (36 patients, 80%) with deflated cuff in 29 patients (64%), followed by ZEEP trials 9 patients (20%) and T-piece trials with inflated cuff 6 (13%). Complications during ICU are shown Tables 2 and 3 and were mainly infections. Number of VAP before the first SBT did not differ between intubated and tracheotomized patients nor between WEAN Safe groups (Table 3). There was no difference on the interval between first SA and successful weaning between patients < 65 years (early- and late-onset) and those aged ≥ 65 years (very-late-onset MG) (Table 4) even when considering subgroups within late-onset MG.

**Table 3:** Patient’s characteristics according to the WEAN safe classification

*N* = 78	No separation attempt *n* = 0	Short wean (24 h) *n* = 2	Intermediate wean (2–6 days) *n* = 31	Prolonged wean (≥ 7 days) *n* = 43	Failed wean *n* = 2	*p* value
Age group						0.500
Age < 65 years-old, n (%)	na	1 (50%)	11 (35%)	17 (40%)	0	
65 < Age < 75 years-old, n (%)	na	0	8 (26%)	9 (21%)	2 (100%)	
75 < Age < 85 years-old, n (%)	na	1 (50%)	7 (23%)	12 (28%)	0	
Age > 85 years-old, n (%)	na	0	5 (16%)	5 (12%)	0	
Cardiac comorbidity, n (%)	na	1 (50%)	17 (55%)	25 (58%)	1 (50%)	0.985
Thymoma, n (%)	na	0	10 (32%)	16 (37%)	1 (50%)	0.689
No immunosuppressive treatment at admission	na	0	10 (32%)	29 (67%)	0	**0.004**
**Ventilation characteristics**						
Time from admission to intubation (days)	na	6 [6–6]	4 [1–8]	4 [0–7]	4 [3–4]	0.970
Time from first separation attempt to successful weaning (days)	na	1 [1–1]	3 [2–4]	17 [10–29]	0	**< 0.001**
Total ventilation (days)	na	14 [14–14]	11 [7–15]	34 [19–52]	31 [23–38]	**< 0.001**
Tracheotomized patients, n (%)	na	0	3 (10%)	29 (67%)	2 (100%)	**< 0.001**
**Complications during ICU stay**						
Ventilator-associated pneumonia before first spontaneous breathing attempt, n (%)						0.942
0	na	2 (100%)	27 (87%)	35 (81%)	2 (100%)	
1	na	0	4 (13%)	7 (16%)	0	
2	na	0	0	1 (2%)	0	
**Outcome**						
Length of stay in ICU, days	na	17 [16–18]	24 [16–40]	43 [31–70]	33 [26–39]	**0.002**
Length of stay in Hospital, days	na	44 [28–59]	27 [18–43]	55 [37–86]	34 [27–41]	**0.002**
Death in ICU, n (%)	na	0	2 (6%)	2 (5%)	2 (100%)	**< 0.001**
Death at 12 months, n (%)	na	0	7 (23%)	6 (14%)	2 (100%)	**0.020**

**Table 4:** Ventilation and weaning modalities according to early and late- or very-late-onset MG

	Total population *n* = 81	Early and late-onset MG (< 65 years) *n* = 31	Very late-onset MG (≥ 65 years) *n* = 50	*p* value
Patients characteristics				
Age (years)	70 [54–81]	49 [36–59]	79 [72–84]	**< 0.001**
Female, n (%)	31 (38%)	16 (52%)	15 (30%)	0.078
Comorbidities, n (%)				
None	23 (28%)	15 (48%)	8 (16%)	**0.002**
Pulmonary disease	15 (19%)	6 (19%)	9 (18%)	0.879
Cardiac disease	46 (57%)	9 (29%)	37 (74%)	**< 0.001**
Renal insufficiency	3 (5%)	1 (4%)	2 (6%)	0.764
Liver disease	3 (4%)	1 (3%)	2 (4%)	0.858
Neoplasm (other than thymoma)	5 (7%)	0	5 (12%)	0.054
Neurological disease	11 (18%)	6 (23%)	5 (14%)	0.377
Other	8 (10%)	6 (19%)	2 (4%)	0.487
AChR antibodies, n (%)	70 (86%)	24 (77%)	46 (92%)	
Thymoma, n (%)	28 (35%)	15 (48%)	13 (26%)	**0.040**
**Reason for intubation**,** n (%)**				
Acute hypoxemic respiratory failure, n (%)	42 (52%)	15 (48%)	27 (54%)	0.494
Acute hypercapnic respiratory failure, n (%)	27 (31%)	11 (35%)	16 (32%)	0.844
*With associated hypoxemia*,* n (%)*^$^	*13 (48%)*	*4 (36%)*	*9 (56%)*	*-*
Other indications, n (%)	12 (15%)	5 (16%)	7 (14%)	-
**Ventilation characteristics**				
Time from admission to intubation (days)	4 [0–6]	2 [0–7]	4 [1–6]	0.726
Time from intubation to first separation attempt (days)	9 [4–21]	10 [6–28]	8 [3–21]	0.823
Time from intubation to tracheotomy (days)	na	19 [12–26]	11 [7–20]	0.193
Time from tracheotomy to first separation attempt (days)	na	11 [5–14]	7 [3–17]	0.527
Time from first separation attempt to successful weaning (days)	7 [3–19]	8 [3–17]	7 [4–21]	0.808
Total ventilation (days)	20 [11–38]	24 [12–38]	18 [11–38]	0.863
Non-invasive ventilation after extubation, n (%)	11 (14%)	5 (16%)	6 (12%)	0.564
Reintubation within 48 h, n (%)	4 (5%)	1 (3%)	3 (6%)	0.577
Reintubation between 48 h and 7 days, n (%)	0	0	0	-
WEAN Safe, *n* = 78				0.690
No separation attempt	0	0	0	
Short wean (<24h)	2 (3%)	1 (3%)	1 (2%)	
Intermediate wean (2–6 days)	31 (40%)	11 (38%)	20 (41%)	
Prolonged wean (≥7 days)	43 (55%)	17 (59%)	26 (53%)	
Failed wean	2 (3%)	0	2 (4%)	
**Complications during ICU stay**				
Ventilator-associated pneumonia before first spontaneous breathing attempt, n (%)				0.440
0	69 (85%)	26 (84%)	43 (86%)	
1	11 (14%)	4 (13%)	7 (14%)	
2	1 (1%)	1 (3%)	0	
Other infections, n (%)	19 (23%)	5 (16%)	14 (28%)	0.202
Hemorrhagic event, n (%)	3 (4%)	1 (3%)	2 (4%)	0.653
Thromboembolic event, n (%)	10 (12%)	3 (10%)	4 (8%)	0.791
Cardiogenic pulmonary edema, n (%)	7 (9%)	1 (3%)	6 (12%)	0.062
Acute kidney injury, n (%)	7 (9%)	1 (3%)	6 (12%)	0.062
**Outcome**				
Length of stay in ICU, days	32 [22–56]	33 [23–55]	32 [22–58]	0.481
Length of stay in Hospital, days	41 [27–65]	40 [28–59]	42 [25–67]	0.364
Death in ICU, n (%)	6 (7%)	0	6 (12%)	**0.045**
Death at 12 months, n (%)	15 (18%)	3 (10%)	12 (24%)	0.107
Myasthenic score at ICU discharge, *n* = 61	80 [66–90]	81 [67–92]	76 [64–89]	0.513
Myasthenic score at 6 months, *n* = 53	90 [82–97]	92 [81–100]	90 [83–95]	0.904
Myasthenic score at 12 months, *n* = 52	94 [80–100]	95 [82–100]	90 [80–96]	0.427

$ corresponds to the number of hypoxemic patients among the hypercapnic patients

Multivariate analysis, which included age, cardiovascular comorbidities, presence of thymoma, absence of any MG-specific immunosuppressive treatment at ICU admission, acute hypercapnic respiratory failure, infectious cause of decompensation and at least one VAP before the first SA found that only thymoma (OR = 3.02 IC95% [1.01–9.07], *p* = 0.0451) and absence of MG-specific immunosuppressive specific at ICU admission (OR = 3.70 IC95% [1.22–11.23], *p* = 0.0156) as associated with prolonged weaning.

### Outcome

Six patients (7%) died in ICU: two from respiratory failure, one from cardiac arrest and three following the withdrawal of life-sustaining therapies in the context of multiple comorbidities. Of these, three had been successfully weaned off invasive ventilation. At 12 months, 15 patients (19%) had died (Table 2). Neurological improvement, as assessed by the Myasthenia Muscle Score at ICU discharge, and at 6 and 12 months, is shown in Fig. 4.

## Discussion

Among 81 first-time admissions for MC, 74 patients (91%) underwent intermediate or prolonged weaning per the WEAN Safe classification. Despite 62% of patients having very-late-onset MG, weaning characteristics did not differ significantly compared to those with MG onset before age 65. In multivariate analysis, only the presence of thymoma and the absence of MG-specific immunosuppressive treatment at ICU admission were associated with prolonged weaning. ICU mortality, tended to be less favorable in the very-late-onset MG though the difference was not statistically significant at one year. Nevertheless, most patients showed substantial neurological recovery, with near-normal myasthenic muscle score at one year (median 94/100 [80–100]).

Few studies on MG in the ICU are available. Most are single-center and involve small cohort [4, 21, 23]. Recently, a large German multicenter retrospective study of 250 MC cases [9] and a French single-center with 126 MC patients were published [11]. Their patients populations are comparable, predominantly featuring late- or very-late-onset MG (67+/-16 in the German study compared to 70 [54–81] in ours), AChR antibody positivity, slight male predominance, a high proportion of inaugural MC (20.8% compared to 32% in our study), thymoma in ~ 30% and multiples comorbidities.

The median ventilation duration in our cohort (20 days [11–38]) was, as expected, longer than that reported for general medical ICU patients (7 days on average [10]) and also longer than durations found in other studies involving myasthenic patients (12–17 days) [4, 11, 21, 24]. This may be due to 42% of patients being transferred from another ICU (likely due to weaning difficulties), older age, and a higher proportion of MuSK antibody-positive patients in our cohort Our results are not comparable to German’s study, as 45% of their patients underwent weaning outside the ICU in rehabilitation or weaning centrs [25] whereas all our patients were weaned within our ICU.

Definitions used to describe the weaning vary across studies. This shortcoming is probably explained by the rarity of the disease and the near absence of MG patients in general ICU weaning studies. This makes direct comparison difficult. We therefore used the WEAN Safe classification. Interestingly, 91% of our cohort fell into the difficult or prolonged weaning categories, compared to just 17% in the WEAN Safe study.

Most of the final SBTs in intubated patients were T-piece trials, consistent with prior MG studies [5, 11]. About one third (28%) of our SBT lasted more than 2 h, with a median of 175 [64–475] minutes, aligning with expert opinions advocating longer SBT durations in MG [5, 26]. However, no consensus exists on optimal duration. In contrast, Mazeraud et al. used 8-hour trials for all patients. Our T-piece trials were mainly conducted with deflated cuff; but due to limited sample size, no comparison was made between deflated and inflated cuff groups. Notably, limited data on cuff status are available in the literature [15, 16, 18, 14].

Eleven patients (14%) received non-invasive ventilation (NIV) immediately after separation. The post-extubation strategy, whether NIV or high-flow nasal oxygen, remains unclear, as neuromuscular patients are often excluded from recent trials and guidelines [27–29]. Small studies suggest NIV may benefit MG patients immediately post-extubation, especially in cases of diaphragmatic fatigue without major bulbar dysfunction [24, 26].

Thirty-four patients (42%) underwent tracheostomy, with a median delay of 13 days [7–22]. Data on tracheostomy are lacking in the German study, and none of the patients in Mazeraud et al.’s study received one. However, our rate aligns with other retrospective studies (20–50%). Among them, 3 patients (9%) required re-ventilation within 48 h post-separation due to clinical worsening (one hypercapnia, two VAPs). No patient required reintubation after decanulation. Interestingly, three patients who underwent early tracheostomy had their first SBT after the procedure, with some achieving weaning in less than 7 days, thus placing them in the intermediate wean group. This might confirm the benefit of early tracheostomy in myasthenic patients to shorten the duration of mechanical ventilation [19].

Previous studies identified factors like infection, low maximal inspiratory pressure, late-onset MG, thymoma and failed NIV as risk factors for prolonged weaning [11, 9]. In our multivariate analysis, only thymoma and absence of MG-specific immunosuppressive treatment at ICU admission were associated.

Extubation failure rate in our cohort was very low (4/81, 5%) versus approximately 14% in general ICU patients, and 26–43% in MG cohorts [9, 21, 22]. These results likely reflect our relatively cautious weaning practice in MG patients, with repeated and prolonged SBTs. While extubation failure increases MV duration and mortality [30, 21], unnecessary prolonged invasive ventilation can also increase complications and ICU length of stay [31]. It is important to note that in our cohort, all patients who experienced EF were ultimately successfully weaned and thus not categorized as weaning failures according to the WEAN SAFE definitions.

The occurrence of VAP before the first spontaneous attempt (15% of patients) was in the range of those reported in the general ICU population (estimated between 5 and 40%) [32] likely due to to prolonged ventilation and immunosuppressive treatments. ICU mortality was 7%, lower than the mortality usually reported for ventilated ICU patients (~ 30%, per Wean Safe) but comparable to the reported mortality in MC (between 4% and 16%) [5, 26]. The causes of death identified were similar to those reported in the literature: respiratory failure, cardiac arrest, and withdrawal of life sustained therapy [25, 33]. Neurological outcomes were good, with MMS improvements at 6 and 12 months. The median MMS at 1 year was 94/100 [80–100], indicating excellent disease control at one-year, and symptom reversibility in most patients.

Neither weaning parameters nor outcome differed significantly between early- and late-onset versus very-late-onset MG, despite more comorbidities in very-late onset MG.

The main limitation of our study is its retrospective design, which limits access to detailed clinical and paraclinical data during ICU stay and at the time of weaning. A second limitation is the small sample size, explained by the rarity of the disease and the single-center nature of the study. In contrast to the definitions used in the WIND and WEAN SAFE studies, we choose to not consider unplanned extubations as SA, and reintubation following such event as EF. This methodological choice may have led to a lower number of recorded SA, and slightly modifying the distribution of patients across weaning categories. This should be considered when comparing our findings to studies using WEAN-safe definitions.

Most patients with MC experienced intermediate or prolonged weaning according to the WEAN safe classification. The presence of thymoma and the absence of MG-specific immunosuppressive treatment at ICU admission were associated with prolonged weaning, whereas age and late-onset disease were not. Given the low incidence of MC requiring invasive mechanical ventilation, even in referral centers, our results underline the urgent need for multicenter, prospective observational studies, including centers with varying levels of expertise, in order to establish more robust and generalizable weaning strategies in this rare but high-risk population.

## Data Availability

The data used during the current study are available from corresponding author on reasonable request.

## Declarations

*Nicolas Weiss perceived consultant fees from Alexion and Owkin*.

## Abbreviations

MG: Myasthenia gravis
MC: Myasthenic crisis
anti-AChR: Acetylcholine receptor antibodies, anti-MuSK, muscle specific kinase antibodies, IV-Ig, intravenous immunoglobulins
TPE: Therapeutic plasma exchange
